# Basic Motor Competencies in Italian Schoolchildren Using the MOBAK‐Test: Normative Data for a Novel Framework

**DOI:** 10.1002/ejsc.70084

**Published:** 2025-11-12

**Authors:** Matteo Giuriato, Alessandro Gatti, Marco Del Bianco, Agnese Pirazzi, Caterina Cavallo, Simone Balconi, Vittoria Carnevale Pellino, Christian Herrmann, Roberto Codella, Matteo Vandoni, Nicola Lovecchio

**Affiliations:** ^1^ Laboratory of Adapted Motor Activity (LAMA) Department of Public Health Experimental Medicine and Forensic Science University of Pavia Pavia Italy; ^2^ National PhD Programme in One Health Approaches to Infectious Diseases and Life Science Research, Department of Public Health, Experimental and Forensic Medicine University of Pavia Pavia Italy; ^3^ Physical Education Research Group Zurich University of Teacher Education Zurich Switzerland; ^4^ Department of Biomedical Sciences for Health University of Milan Milan Italy; ^5^ Department of Human and Social Science University of Bergamo Bergamo Italy

**Keywords:** basic motor competencies, motor development, physical education evaluation

## Abstract

Basic motor competencies (BMC) are foundational skills essential for children's participation in sports and physical activities. While normative data exist in countries like Germany and Switzerland, benchmarks for Italian children are lacking. This study addresses this gap by establishing normative data for Italian schoolchildren aged 5–11 years using the MOBAK test battery, which assesses self‐movement (balancing, rolling, jumping, running) and object movement (throwing, catching, bouncing, dribbling) competencies. A total of 1626 children (838 boys, 788 girls) from kindergarten to 10–11 years in Northern Italy were assessed using the MOBAK tests battery. Descriptive statistics, percentile ranks, z‐scores, and T‐scores were used to establish normative values, while analysis of variance (ANOVA) examined differences by sex and grade level. The normative data and the comparison between sexes highlight developmental trends in motor skills, with raw scores increasing steadily across age groups. Boys consistently outperformed girls in object movement tasks, while girls excelled in self‐movement tasks, particularly in grades 5 and 6. Notably, boys had increasing difficulty in achieving high scores with age, as seen in the changing percentile ranks for the same raw scores. In contrast, girls exhibited more stable development, maintaining or improving percentile ranks over time. This study provides the first normative data for BMC in Italian children, highlighting sex‐based differences and developmental trajectories. The benchmarks offer a vital tool for educators and policymakers to design tailored interventions, supporting children's motor competence and promoting lifelong physical activity.

## Highlights

- This study establishes normative data for Basic Motor Competencies (BMC) in Italian schoolchildren aged 5–11 years, utilizing the MOBAK test battery.
- The findings reveal consistent developmental trends in BMC, with boys excelling in object movement tasks and girls in self‐movement tasks.
- The normative data provided offer educators and policymakers critical tools to design interventions that promote physical activity and address developmental disparities.

## 1 Introduction

Motor competence is an umbrella term that refers to different classes of human movement and motor skills, often defined differently depending on the researcher's background or preference. These definitions include terms such as *fundamental movement skills*, *motor performance*, physical fitness, and *motor proficiency* (Logan et al. 2018). While these definitions describe movements involving fine and gross motor skills, motor coordination, and whole‐body movement (Robinson et al. 2015), a sub‐concept of basic motor competencies (BMC) has recently emerged (Scheuer et al. 2019). BMC represent the essential motor competencies that children and adolescents must develop to participate effectively in sports and exercise activities (Herrmann, Bund, et al. 2015). These competencies form the foundation for acquiring specific sports skills and techniques and can be assessed through observable motor performance (Kurz et al. 2008). Furthermore, as highlighted by Hulteen et al. (2018), BMC are crucial bases for engaging in different physical activities. The model proposed by the authors underscores how the development of these competencies supports physical, psychological, and social health throughout the lifespan, strengthening the importance of early assessment and promotion of BMC in educational contexts.

Indeed, BMC represent the essential motor competencies that children and adolescents should develop to effectively participate in sports and exercise activities (Herrmann, Bund, et al. 2015) and become robust in context‐specific scenarios (e.g., throwing a ball at a target) (Herrmann et al. 2015; Herrmann and Seelig 2017). Consequently, over time, it has become crucial for teachers, trainers, and coaches to assess these skills in ways that differ from those used in the 1980s and 1990s. During that period, motor assessment was largely confined to evaluating physical fitness such as strength and speed, or using tests based on quantitative dimensions (Bös and Schlenker 2011).

Moreover, the evaluation of motor competencies must be tailored to the specific grade and age of the child, with an emphasis on achieving the desired movement outcome. This approach is essential because it is based on the understanding that the maturation of the central nervous system is a key factor influencing motor skill development (Barnett et al. 2022; Herrmann and Seelig 2017; Robinson et al. 2015).

In light of this, the *Motorische Basiskompetenzen* (MOBAK) test battery (Herrmann 2018; Herrmann and Seelig 2020) provides a standardized and reliable framework for assessing BMC in children, from preschool to primary school age, including product‐oriented evaluations.

Several countries (e.g., Slovakia, Switzerland, Germany, Belgium, Chile) have evaluated BMC using the MOBAK test (Quintriqueo‐Torres et al. 2022; Wälti et al. 2022). For the Italian population, to the best of our knowledge, only two studies have reported data on the MOBAK test. Specifically, a cross‐sectional study on children aged 8–10 years (*n* = 282) by Wälti et al. (2023), and a pilot intervention study using the MOBAK‐5 test in a small sample by Monacis et al. (2022). However, neither of the two studies aimed to establish normative reference values. Normative values are statistical benchmarks derived from large population samples, enabling the interpretation of individual test scores relative to specific peer groups (e.g., by age, sex, or country). In the context of the MOBAK tests (Herrmann 2018; Herrmann and Seelig 2020), such norms are crucial for identifying age‐ and sex‐specific levels of basic motor competence among schoolchildren in Northern Italy. These normative references might support physical education teachers and coaches in assessing and comparing BMC levels both nationally and internationally. Providing national norm value tables ensures the cultural and educational relevance of interpreting test scores within the Italian context, as demonstrated in this study.

For example, a study in Switzerland found that, using MOBAK‐test in preschool‐children, boys performed better in tasks involving objects, whereas girls achieved better scores in self‐movements tasks. Additionally, older children seemed to perform better than their younger peers (Herrmann et al. 2019, 2021). Similarly, boys in Slovakia, between third and fourth grade, were found to be more proficient in object‐control tasks (e.g., throwing, grasping, bouncing, and dribbling) compared to girls (Šiška et al. 2024). Conversely, in the same study (Šiška et al. 2024), girls outperformed boys in balance and jumping skills.

This trend has also been confirmed in European and Chilean studies using the MOBAK 5–6 test for children in fifth and sixth grades (Carcamo‐Oyarzun and Herrmann 2023; Quintriqueo‐Torres et al. 2022; Wälti et al. 2022, 2023). In self‐movement tasks, girls in Chile scored similarly to boys (Quintriqueo‐Torres et al. 2022), while girls in Europe appeared to outperform boys (Wälti et al. 2022).

Given the pivotal role of assessing BMC within physical education (PE) context, this study aims to assess BMC among children aged 5–11 years in Italy using the MOBAK testing batteries. Additionally, it seeks to establish normative values for the MOBAK‐KG, MOBAK 1–2, MOBAK 3–4, and MOBAK 5–6 batteries for the Italian population. To this end, a cross‐sectional study was conducted involving a sample of school‐aged children, with data gathered through standardized motor assessments (MOBAK battery test) in school settings.

Specifically, for preschool children (5.36 years ± 0.41) we used the MOBAK‐KG; MOBAK 1–2 was administered to students in their first (6.44 years ± 0.38) and second (7.36 years ± 0.32) grade of elementary school, MOBAK 3–4 to students of third (8.39 years ± 0.39) and fourth (9.37 years ± 0.41) grade elementary school, MOBAK 5–6 to children of fifth grade of elementary school (10.43 years ± 0.33) and class 6 (11.59 years ± 0.40) of middle school.

## 2 Material and Methods

### 2.1 Participants

We conducted a study involving 1626 children (788 girls and 838 boys) from preschool to the class 6 in the province of Monza, located in Northwestern Italy.

The participants were evaluated during their curricular PE classes by sports specialists from the Universities of Pavia, Bergamo, and Milan, using the MOBAK test, which consists of eight assessments measuring BMC (Carcamo‐Oyarzun and Herrmann 2020).

Ninety children performed the MOBAK‐KG test (Girls = 44; 48%), 616 performed the MOBAK 1–2 test (Girls = 288, 47%), 615 performed the MOBAK 3–4 test (Girls = 301, 49%), and 392 performed the MOBAK 5–6 test (Girls = 200, 51%). The children performed the tests according to the MOBAK protocol, following a detailed explanation and a single demonstration. Additionally, specialists provided assistance during the execution of the tests without influencing the children's performance.

The inclusion criteria were children aged 5–11 years, of both genders, with no known cardiovascular, respiratory, or neurological diseases, and without developmental disorders, learning disabilities, or psychosis that might affect growth or school performance. The exclusion criteria included comorbidities, reported orthopedic injuries in the past 6 months, and any condition that contraindicated participation in curricular PE classes. All participants had knowledge of the Italian language.

Written informed consent was obtained from parents or legal guardians after the study procedures were explained. Children were informed at school that their participation was voluntary and that they could withdraw at any time by verbally communicating their absence, without any negative consequences. No academic credit or benefit was offered for participation. The study protocol was approved by the CET 6 Lombardia (Comitato Etico Territoriale Lombardia 6; Prot. *n*. 25557/25)

### 2.2 Basic Motor Competence

The MOBAK protocol assesses eight BMC items, which are divided into two motor areas: object movement and self‐movement. Each area includes four tasks designed to evaluate different aspects of motor competence (Herrmann 2018).

The object movement area was divided into four tasks: throwing, catching, bouncing, and dribbling, while the self‐movement area consisted of balancing, rolling, jumping, and running. Detailed description of the tasks the specific criteria for scoring have been described elsewhere and in the supplementary materials (Herrmann 2018; Herrmann et al. 2019; Herrmann and Seelig 2017).

The MOBAK protocol includes a series of developmental tests adapted for different school grades. MOBAK KG is designed for preschool children; MOBAK 1–2 for children in the first and second grades of elementary school; MOBAK 3–4 for children in the third and fourth grades of elementary school; and MOBAK 5–6 for children in the fifth grade of elementary school and first grade of middle school in Italian scholastic system (class 6).

Each MOBAK‐item can be scored between 0 and 2 points, with a maximum of 8 points for each area (object movement and self‐movement), and a maximum total score of 16 points to assess overall BMC. Based on this, a total MC category was created with a maximum possible score of 16 points (Total Score). The MOBAK battery has shown good reliability indices in different studies. For MOBAK 1–2, internal consistency was measured with a Cronbach's alpha of 0.79, while MOBAK 3–4 reported an alpha of 0.83. Test‐retest reliability, which measures stability over time, showed values between 0.74 and 0.85 for different age groups. MOBAK KG's latent factor reliability was 0.80, indicating good reliability, while for MOBAK 5–6, factor reliabilities were satisfactory: the self‐movement subscale had a factor reliability (FR) of 0.59, and object movement had an FR of 0.85 (Herrmann 2018; Herrmann et al. 2019; Herrmann and Seelig 2017).

The children performed the MOBAK tests according to their school class, following a description and a single demonstration of each task. For the throwing and catching tasks, the children were given six attempts per item, with the results scored as follows: 0–2 successful hits = 0 points, 3–4 hits = 1 point, and 5–6 hits = 2 points. For the remaining tasks, bouncing, dribbling, balancing, rolling, jumping, and running, each child had two attempts per item. The scoring was as follows: two failed attempts = 0 points, one successful attempt = 1 point, and two successful attempts = 2 points. A team of eight test administrators, all graduates in sports science, ensured that the tests were administered in a standardized manner and that the scores were recorded reliably and accurately.

### 2.3 Anthropometric Measures

All the anthropometric measures were taken by a sport specialist before the start of any session. Weight was measured by standing in lightweight clothing in the center of a scale (Seca GmbH & Co. KG, Hamburg, Germany) with hands at the sides and looking straight ahead in the recorder direction. Standing height was assessed using a Harpenden stadiometer (Holtain Ltd., Cross‐well, Crymych, UK) with a fixed vertical table and an adaptable head (Norton and Eston 2018). Then Body Mass Index (BMI) was computed by dividing the weight (kilograms) by the height^2^ (meters squared).

### 2.4 Statistical Analysis

The establishment of age‐ and sex‐specific normative values for motor competence is essential during childhood and adolescence, as it enables the early detection of developmental delays and supports the design of equitable, targeted interventions to promote lifelong physical activity and health (Barnett et al. 2016). We calculated the minimum number of participants required per grade level to estimate normative values for the MOBAK total score (0–16 scale), ensuring a 95% confidence interval with a half‐width of ±1 raw score point. Using variance data from a previously published study (Legarra‐Gorgoñon et al. 2023), we applied a precision‐based method and determined that each subgroup needed at least 59 children.

To assess the normative values we calculated the mid‐interval percentile rank (PR), the z‐score and the equivalent T‐score derivable from it (T; with a mean value of 50 and a standard deviation of 10), following the methods used in previous studies (Herrmann 2018; Herrmann et al. 2019). This standardization is based on the raw‐score distributions in the present sample.

The percentile rank indicates what percentage of the children in the present sample achieved a performance comparable to or lower than that of the tested child. The clarity of this value makes it suitable for communicating the results to parents or (kindergarten) teachers. The T‐value (*M* = 50, SD = 10) makes it possible to assess whether the performances achieved by the child are below or above average in comparison with the standardization sample. Values in the domain of one standard deviation below and above the mean (40 ≤ T‐value ≤ 60) are achieved by two‐thirds of all children and are regarded as average performances.

To compare the differences between sexes divided by grade, we performed an analysis of variance (ANOVA). Firstly, we performed a Shapiro‐Wilk test to assess the normality of the data. Then if the data were parametric (Shapiro‐Wilk *p*‐value > 0.05) a type‐I one‐way ANOVA, or if non‐parametric (Shapiro‐Wilk *p*‐value > 0.05) a Krustal‐Wallis ANOVA.

All analyses were conducted using R software, version 4.4.0 (R Foundation for Statistical Computing).

## 3 Results

Descriptive statistics was presented in Table 1. To create normative values for the Italian population, we assigned a mid‐interval percentile rank (PR), z‐scores and a T‐value to the raw values achieved by the children in the total MOBAK score (sums between object movement domain and self‐movement domain; Table 2).

**TABLE 1:** Descriptive characteristics of the total sample divided by sex.

	All (*n* = 1626)	Boys (*n* = 838)	Girls (*n* = 788)
Age (years)	8.11 (8.03, 8.20)	8.04 (7.93, 8.16)	8.19 (8.06, 8.31)
Weight (kg)	31.18 (30.70, 31.65)	31.20 (30.54, 31.85)	31.16 (30.47, 31.86)
Height (cm)	1.33 (1.33, 1.34)	1.33 (1.33, 1.34)	1.33 (1.32, 1.34)
Body mass index z‐score	0.47 (0.41, 0.54)	0.54 (0.45, 0.63)	0.40 (0.32, 0.49)
*MOBAK*			
Object movement score	4.47 (4.36, 4.59)	5.18 (5.04, 5.33)	3.72 (3.56, 3.87)
Self‐movement score	4.41 (4.31, 4.52)	4.22 (4.07, 4.36)	4.62 (4.47, 4.77)
Total score	8.88 (8.70, 9.07)	9.40 (9.16, 9.65)	8.33 (8.07, 8.60)

*Note:* Data are presented as mean (95% confidence interval).

**TABLE 2:** Normative Values of the MOBAK Score (percentile ranks, *z*‐score and t‐values) divided by Grade and sex.

	MOBAK KG							
	Boys (*n* = 39)				Girls (*n* = 37)			
	RS	PR	*Z*	*T*	RS	PR	*Z*	*T*
KG	0	—	—	—	0	—	—	—
	1	—	—	—	1	—	—	—
	2	—	—	—	2	—	—	—
	3	0	—	—	3	—	—	—
	4	5	−1.62	34	4	0	—	—
	5	11	−1.25	37	5	3	−1.91	31
	6	13	−1.12	39	6	6	−1.59	34
	7	21	−0.80	42	7	17	−0.97	40
	8	29	−0.55	44	8	22	−0.77	42
	9	39	−0.27	47	9	42	−0.21	48
	10	42	−0.20	48	10	67	0.43	54
	11	53	0.07	51	11	78	0.76	58
	12	66	0.41	54	12	89	1.22	62
	13	76	0.72	57	13	94	1.59	66
	14	84	1.00	60	14	—	—	—
	15	97	1.94	69	15	100	—	—
	16	—	—	—	16	—	—	—
	**MOBAK 1–2**							
	**Boys (*n* = 163)**				**Girls (*n* = 117)**			
	**RS**	**PR**	***Z***	***T***	**RS**	**PR**	***Z***	***T***
Grade 1	0	—	—	—	0	—	—	—
	1	0	—	—	1	—	—	—
	2	—	—	—	2	0	—	—
	3	—	—	—	3	1	−2.38	26
	4	1	−2.50	25	4	3	−1.82	32
	5	2	−2.09	29	5	8	−1.42	36
	6	5	−1.65	33	6	11	−1.22	38
	7	14	−1.07	39	7	19	−0.88	41
	8	17	−0.94	41	8	27	−0.62	44
	9	27	−0.63	44	9	36	−0.35	46
	10	31	−0.48	45	10	53	0.09	51
	11	42	−0.20	48	11	68	0.47	55
	12	54	0.09	51	12	80	0.85	58
	13	75	0.66	57	13	89	1.22	62
	14	86	1.10	61	14	96	1.72	67
	15	91	1.36	63	15	100	—	—
	16	96	1.71	67	16	—	—	—
	**Boys (*n* = 136)**				**Girls (*n* = 149)**			
	**RS**	**PR**	***Z***	***T***	**RS**	**PR**	***Z***	***T***
Grade 2	0	—	—	—	0	—	—	—
	1	—	—	—	1	—	—	—
	2	—	—	—	2	0	—	—
	3	—	—	—	3	1	−2.47	25
	4	—	—	—	4	—	—	—
	5	0	—	—	5	2	−2.21	28
	6	1	−2.44	26	6	5	−1.67	33
	7	2	−2.18	28	7	6	−1.61	34
	8	6	−1.57	35	8	9	−1.31	47
	9	14	−1.09	39	9	16	−1.01	40
	10	20	−0.83	42	10	24	−0.72	43
	11	28	−0.60	44	11	34	−0.42	46
	12	47	−0.07	49	12	45	−0.12	49
	13	63	0.33	53	13	59	0.22	52
	14	82	0.91	59	14	78	0.79	58
	15	92	1.41	64	15	91	1.31	63
	16	98	2.02	70	16	99	2.21	72
	**MOBAK 3–4**							
	**Boys (*n* = 156)**				**Girls (*n* = 143)**			
	**RS**	**PR**	***Z***	***T***	**RS**	**PR**	***Z***	***T***
Grade 3	0	0	—	—	0	0	—	—
	1	1	−2.49	25	1	4	−1.73	33
	2	3	−1.85	32	2	8	−1.42	36
	3	9	−1.34	37	3	13	−1.11	39
	4	13	−1.13	39	4	20	−0.83	42
	5	17	−0.97	40	5	27	−0.60	44
	6	23	−0.75	42	6	34	−0.42	46
	7	29	−0.55	45	7	38	−0.30	47
	8	45	−0.14	49	8	49	−0.02	50
	9	57	0.19	52	9	62	0.30	53
	10	65	0.39	54	10	69	0.50	55
	11	74	0.65	56	11	75	0.66	57
	12	81	0.89	59	12	85	1.02	60
	13	86	1.07	61	13	90	1.29	63
	14	91	1.34	63	14	96	1.73	67
	15	96	1.77	68	15	98	2.03	70
	16	97	1.94	69	16	—	—	—
	**Boys (*n* = 149)**				**Girls (*n* = 167)**			
	**RS**	**PR**	***Z***	***T***	**RS**	**PR**	***Z***	***T***
Grade 4	0	0	—	—	0	0	—	—
	1	1	−2.47	25	1	1	−2.20	28
	2	3	−2.05	30	2	2	−1.81	32
	3	7	−1.67	33	3	5	−1.48	35
	4	10	−1.49	35	4	7	−1.29	37
	5	15	−1.27	37	5	10	−1.05	40
	6	20	−1.04	40	6	15	−0.83	42
	7	31	−0.93	41	7	18	−0.50	45
	8	38	−0.53	45	8	30	−0.29	47
	9	46	−0.35	47	9	36	−0.10	49
	10	62	−0.07	49	10	47	0.29	53
	11	73	0.26	53	11	60	0.60	56
	12	80	0.55	56	12	71	0.83	58
	13	88	0.91	59	13	82	1.18	62
	14	96	1.04	60	14	85	1.73	67
	15	98	1.49	65	15	93	2.03	70
	16	99	2.05	70	16	98	2.46	75
	**MOBAK 5–6**							
	**Boys (*n* = 108)**				**Girls (*n* = 98)**			
	**RS**	**PR**	***Z***	***T***	**RS**	**PR**	***Z***	***T***
Grade 5	0	0	—	—	0	0	—	—
	1	3	−1.91	31	1	4	−1.74	33
	2	6	−1.51	35	2	7	−1.46	35
	3	7	−1.44	36	3	14	−1.06	39
	4	14	−1.08	39	4	27	−0.62	44
	5	21	−0.79	42	5	41	−0.22	48
	6	27	−0.61	44	6	51	0.01	50
	7	36	−0.35	47	7	62	0.30	53
	8	46	−0.11	49	8	66	0.41	54
	9	51	0.04	50	9	72	0.59	56
	10	67	0.45	54	10	81	0.89	59
	11	76	0.70	57	11	88	1.16	62
	12	84	1.00	60	12	95	1.63	66
	13	89	1.22	62	13	96	1.74	67
	14	91	1.32	63	14	97	1.87	69
	15	96	1.78	68	15	—	—	—
	16	99	2.35	74	16	—	—	—
	**Boys (*n* = 92)**				**Girls (*n* = 100)**			
	**RS**	**PR**	***Z***	***T***	**RS**	**PR**	***Z***	***T***
Grade 6	0	0	—	—	0	0	—	—
	1	2	−1.98	30	1	4	−1.75	33
	2	5	−1.66	33	2	9	−1.34	37
	3	7	−1.46	35	3	15	−1.03	40
	4	11	−1.23	38	4	23	−0.73	43
	5	16	−1.01	40	5	33	−0.43	46
	6	22	−0.78	42	6	40	−0.24	48
	7	39	−0.29	47	7	56	0.14	51
	8	45	−0.14	49	8	68	0.46	55
	9	65	0.39	54	9	77	0.73	57
	10	80	0.82	58	10	82	0.91	59
	11	86	1.06	61	11	89	1.22	62
	12	90	1.30	63	12	96	1.75	67
	13	96	1.80	68	13	98	2.05	70
	14	99	2.26	73	14	—	—	—
	15	100	—	—	15	—	—	—
	16	—	—	—	16	—	—	—

*Note:* Gray shaded: 40 ≤ T‐value ≤ 60.

Abbreviations: PR = percentile rank; RS = raw scores; *T* = T‐value; *Z* = z‐score.

For boys, in kindergarten a raw score of 8 corresponds to a percentile rank of 29, while in Grades 5–6, the same raw scores (RS) of 8 corresponds to a PR of 46. For girls, in kindergarten, the 25th percentile is reached with an RS of 8, with a PR of 22. By Grades 5–6, the 25th percentile is associated with an RS of 9, but the PR has increased to 72.

**FIGURE 1:**
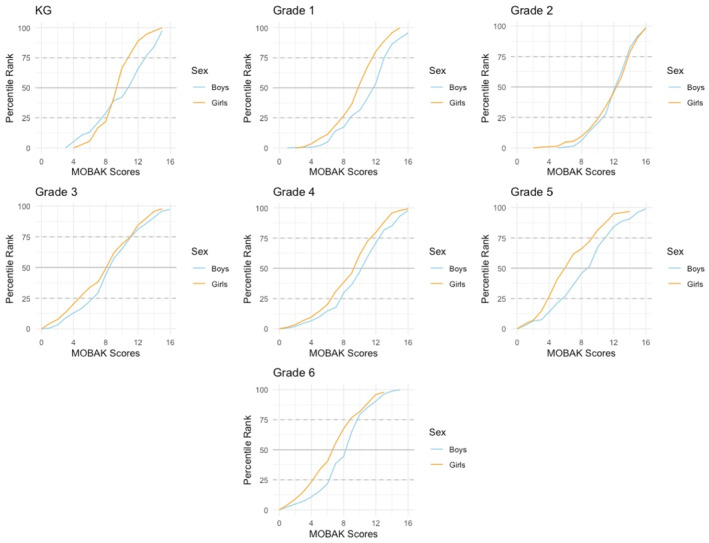
Centile curves for the total MOBAK scores divided by grades and sex.

To facilitate the visualization of the percentile ranks, we represented them graphically through centile curves in Figure 1.

Table 3 shows that boys consistently outperformed girls in object‐movement across all grades (*p* < 0.002). For self‐movement, no sex differences were found in most grades, except in grade 6 and 2 where girls scored higher than boys (*p* < 0.001). In the overall MOBAK scores, boys generally had higher scores than girls, with differences in grades 1, 4, 5, and six (*p*‐value < 0.011), while no differences were observed in KG, grade 2 and 3.

**TABLE 3:** Comparison of Object‐Movement, Self‐Movement, and MOBAK‐score between boys and girls.

	Boys	Girls	η^2^	*p*‐value
Object‐movement skills				
KG	5.26 (4.59, 5.92)	4.00 (3.43, 4.57)	0.08	**0.011**
Grade 1	5.54 (5.26, 5.82)	4.14 (3.80, 4.47)	0.13	**> 0.001**
Grade 2	6.32 (6.06, 6.58)	5.35 (5.06, 5.64)	0.08	**> 0.001**
Grade 3	4.79 (4.44, 5.15)	3.54 (3.17, 3.91)	0.07	**> 0.001**
Grade 4	5.43 (5.11, 5.75)	4.08 (3.73, 4.43)	0.09	**> 0.001**
Grade 5	4.30 (3.86, 4.73)	2.31 (1.92, 2.69)	0.17	**> 0.001**
Grade 6	4.01 (3.55, 4.47)	1.80 (1.42, 2.18)	0.23	**> 0.001**
Self‐movement skills				
KG	4.51 (3.89, 5.14)	5.00 (4.44, 5.56)	0.00	0.302
Grade 1	5.07 (4.80, 5.35)	5.00 (4.68, 5.32)	0.00	0.715
Grade 2	5.19 (4.91, 5.48)	6.01 (5.73, 6.29)	0.06	**> 0.001**
Grade 3	3.38 (3.06, 3.71)	3.83 (3.47, 4.19)	0.01	0.078
Grade 4	4.02 (3.67, 4.37)	4.30 (3.96, 4.63)	0.00	0.226
Grade 5	3.63 (3.18, 4.08)	3.77 (3.30, 4.23)	0.00	0.667
Grade 6	3.46 (3.00, 3.93)	4.37 (3.92, 4.82)	0.04	**0.001**
MOBAK score				
KG	9.77 (8.64, 10.90)	9.00 (8.22, 9.78)	0.0167	0.266
Grade 1	10.61 (10.14, 11.08)	9.14 (8.62, 9.65)	0.0579	**> 0.001**
Grade 2	11.52 (11.13, 11.91)	11.36 (10.91, 11.80)	−0.0035	0.982
Grade 3	8.18 (7.59, 8.77)	7.37 (6.72, 8.02)	0.0046	0.124
Grade 4	9.45 (8.87, 10.03)	8.38 (7.80, 8.96)	0.0191	**0.011**
Grade 5	7.93 (7.19, 8.66)	6.07 (5.36, 6.78)	0.0560	**> 0.001**
Grade 6	7.48 (6.80, 8.15)	6.17 (5.51, 6.83)	0.0361	**0.006**

*Note:* Data are presented as mean (95% confidence interval). Two‐sided *p* and partial eta squared (η^2^) values were obtained from analysis of variance evaluating the differences between sexes. Significance threshold was set at < 0.05. Bold *p*‐values indicate significant differences between the two groups.

## 4 Discussion

This study aimed to provide normative values for the Italian population, from preschool age up to the first year of secondary school, using the MOBAK test. Even if this was not the first attempt to extend the MOBAK to the Italian population (Lovecchio et al. 2024; Monacis et al. 2022; Wälti et al. 2023), our study is the first to assess normative values of the MOBAK test across all grades for the Italian population of both sexes. A prerequisite for targeted motor development is that teachers are aware of the motor competence of children in different age groups. Based on this, they can adapt their educational approach. The standard values developed in our article provide an initial guide to the BMC level of Italian children (Herrmann, Bund, et al. 2015).

The results indicate that boys consistently outperform girls in object movement (see supplementary materials) across all grades. For self‐movement, no significant differences were observed between sexes, except in the second and sixth grades, where girls scored higher (see supplementary materials). These findings are in line with previous Italian studies (Lovecchio et al. 2024; Monacis et al. 2022; Wälti et al. 2023) and gain additional value as they provide a useful evaluating tool for Italian PE teachers. The purpose of providing reference values is not only to describe typical performance by age and sex, but also to allow practitioners to identify children performing below expected levels. The observed differences emphasize the need for sex‐specific values and contextualized interpretation of BMC, especially in a national context such as Italy, where reference data are currently unavailable.

In terms of overall MOBAK scores, boys performed better than girls in the first, fourth, fifth, and sixth grades, whereas no significant differences were found in preschool (KG), second, and third grades. Findings from various studies reveal a consistent trend favoring boys, particularly during early childhood and school‐age periods. Longitudinal and cross‐sectional studies have shown that boys tend to develop superior object movement compared to girls. This disparity appears in early childhood and becomes more pronounced during school‐age years, especially in activities like throwing and kicking, where boys demonstrate greater strength and coordination (Barnett et al. 2016; Logan et al. 2018). Beyond biological differences, gender‐related socialization plays a key role in shaping motor competence trajectories. Robinson et al. (2015), showed that social, cultural, and environmental factors, such as gender stereotypes, parental expectations, and access to structured physical activity, can significantly influence the types of motor experiences children are exposed to.

Boys are typically encouraged to participate in sports and games that involve object control (e.g., football, basketball), which promotes the development of these skills. In contrast, girls may have less exposure to such activities, often receiving encouragement toward other forms of play (Chalabaev et al. 2013).

Indeed, Herrmann and Seelig (2020) demonstrated that boys performed better in object movement, while girls exhibited superior results in self‐movement. Similarly, Carcamo‐Oyarzun and Herrmann (2023) reported differences in BMC between the sexes, highlighting the distinct developmental pathways of motor competence in boys and girls. These findings underscore the necessity for gender‐specific approaches in PE and training.

A similar trend is observed for girls, though with a different dynamic. The results indicate that girls either maintain or improve their motor competence proficiency relative to boys as they age, often displaying better performance in later grades. Notably, there were no statistically significant differences between boys and girls in self‐movement skills from kindergarten through fourth grade. However, in grades five and six, girls outperformed boys, suggesting a potential for greater retention or development of BMC over time. However, the level of BMC in both object movement and self‐movement skills is influenced by age, especially in third‐ and fourth‐grade children (Šiška et al. 2024). Despite differing perspectives on this topic, Scheuer et al. (2019) observed that age has a limited impact on the level of BMC. However, Carcamo‐Oyarzun and Herrmann (2020), found a significant effect of age on object control, with a minor impact on autonomous movements. Conversely, Strotmeyer et al. (2021) did not confirm any significant effect of age on autonomous movement activities. Given these varied findings, PE teachers need to assess children's learning outcomes (Allal 2020), as early identification of motor or learning delays can help improve teaching approaches.

The gap between boys and girls may not solely reflect biological differences but also indicate the need for tailored strategies that address the specific challenges faced by girls in PE. While boys generally perform better in motor tasks, it is crucial to ensure that both sexes have equal opportunities to develop motor competence.

The MOBAK test is a valuable tool for identifying children who may have delayed BMC. Early detection allows for targeted interventions to improve these skills, supporting overall health and development (Herrmann, Bund, et al. 2015). Developing BMC is essential for meeting the diverse demands of sports and exercise (Barnett et al. 2016).

Recent research by Wälti et al. (2022) has revealed notable discrepancies in BMC across different European countries. In terms of object movement, the Salzburg region of Austria shows the highest scores for both males (6.18) and females (4.99). Italy occupies an intermediate position, with scores of 5.36 for males and 4.25 for females, indicating that while the country's performance is not on par with European leaders, the BMC of young Italians are relatively robust. However, there is still room for improvement.

In self‐movement tasks, the Groningen region in the Netherlands stands out for females, with a score of 6.31, followed closely by Zurich, Switzerland, at 6.27. These results could underscore the importance of educational and cultural contexts in shaping perceptions and the development of motor skills. Italy, with a score of 4.71, ranks in the middle, showing lower results than leading countries but still demonstrating a sufficiently positive performance. The MOBAK test instrument evaluates two main domains: object movement and self‐movement competencies, from early childhood through the first years of middle school. The findings offer a comprehensive understanding of motor competence levels in children, particularly regarding gender differences as assessed by MOBAK.

The MOBAK framework adopts a comprehensive pedagogical approach, with a specific emphasis on supporting the development of basic motor competencies. It is important to note that it goes beyond just learning how to move and also includes developing the desire to do things and the ability to interact with other people. This multidimensional approach aligns with the evolving international discussion on physical literacy. This topic advocates for a comprehensive understanding of physical activity that incorporates competence, confidence, and meaningful engagement throughout one's life (Niehues et al. 2024).

However, there are limitations to consider before interpreting our results. Firstly, only a limited number of covariates were considered, which reflects the study's primary aim of providing normative reference values rather than exploring explanatory factors. The study was conducted in a specific geographic and cultural context, which may restrict the generalizability of the results to other populations. Future research would benefit from cross‐cultural comparisons to better understand how different environments and habits influence the development of motor skills in boys and girls. Another limitation is that the kindergarten normative values should be interpreted with caution, as the sample size was insufficient to achieve the desired statistical power. Although the data were collected across different schools in Northern Italy, information on the geographic distribution, contextual characteristics of the schools and socioeconomic information's of the families participating in the study were not recorded, which may limit the interpretation of potential influences related to socioeconomic status.

Additionally, since MOBAK primarily assesses BMC, it would be valuable to explore how these skills translate into more complex motor tasks as children grow. This could be achieved through longitudinal studies, providing a deeper understanding of BMC development over time and the factors contributing to these changes.

## 5 Conclusions

Our study provides the first normative data on the MOBAK test for the Italian population. The discrepancies observed in MOBAK test results across Europe are not only attributable to biological and psychological factors; they are also influenced by environmental, economic, cultural, and public‐administration variables. Italy's intermediate results indicate a position with both potential for growth and opportunities for optimizing current practices. Implementing targeted interventions could strengthen self‐movement‐related BMC in young people, further enhancing motor skill development.

## Funding

The authors have nothing to report.

## Ethics Statement

CET 6 Lombardia (Comitato Etico Territoriale Lombardia 6; prot. *n*. 0025557/25)

## Consent

Written informed consent was obtained from parents or legal guardians after the study procedures were explained. Children were informed at school that their participation was voluntary and that they could withdraw at any time by verbally communicating their absence, without any negative consequences.

## Conflicts of Interest

The authors declare no conflicts of interest.

## Supporting information


Supporting Information S1

## Data Availability

The data that support the findings of this study are available from the corresponding author, upon reasonable request.
