# Supplementary material for: Basic Motor Competencies in Italian Schoolchildren Using the MOBAK‐Test: Normative Data for a Novel Framework
Source: Eur J Sport Sci. 2025 Nov 12;25(12):e70084. doi: 10.1002/ejsc.70084 (PMC12611270; doi:10.1002/ejsc.70084)
Supplement: Supplementary file 1 — Supporting Information S1 [file EJSC-25-e70084-s001.docx]

**Basic Motor Competencies in Italian Schoolchildren Using the MOBAK-test: Normative Data for a Novel Framework**

**Supplementary materials**

**Table S1.** Object-movement domain for KG, 1-2, 3-4 and 5-6 normative values (percentile ranks, z-score and t-values) divided by Grade and sex.

| MOBAK KG | | | | | | | | | | | | | | | | | | | | |  |  |  |  |
| --- | --- | --- | --- | --- | --- | --- | --- | --- | --- | --- | --- | --- | --- | --- | --- | --- | --- | --- | --- | --- | --- | --- | --- | --- |
|  | | Boys (n=39) | | | | | | Girls (n=37) | | | | | | | | | | | |  |  |  |  |  |
|  | | RS | | PR | | Z | T | | | RS | | PR | | | Z | | | T | | | | | | |
| Class KG | | 0 | | - | | - | - | | | 0 | | - | | | - | | | - | | | | | | |
|  |  | 1 | | - | | - | - | | | 1 | | 0 | | | - | | | - | | | | | | |
|  |  | 2 | | 0 | | - | - | | | 2 | | 11 | | | -1.22 | | | 58 | | | | | | |
|  |  | 3 | | 11 | | -1.25 | 37 | | | 3 | | 17 | | | -0.97 | | | 40 | | | | | | |
|  |  | 4 | | 24 | | -0.72 | 43 | | | 4 | | 42 | | | -0.21 | | | 48 | | | | | | |
|  |  | 5 | | 45 | | -0.13 | 49 | | | 5 | | 64 | | | 0.36 | | | 54 | | | | | | |
|  |  | 6 | | 53 | | 0.07 | 51 | | | 6 | | 81 | | | 0.86 | | | 59 | | | | | | |
|  |  | 7 | | 68 | | 0.48 | 55 | | | 7 | | 94 | | | 1.59 | | | 66 | | | | | | |
|  |  | 8 | | 82 | | 0.90 | 59 | | | 8 | | - | | | - | | | - | | | | | | |
| MOBAK 1-2 | | | | | | | | | | | | | | | | | | | | |  |  |  |  |
|  | | Boys (n=163) | | | | | | Girls (n=117) | | | | | | | | | | | |  |  |  |  |  |
|  | | RS | | PR | | Z | T | | | RS | | PR | | Z | | | T | | | | | |  |  |
| Class 1 | | 0 | | 0 | | - | - | | | 0 | | 0 | | - | | | - | | | | | |  |  |
|  |  | 1 | | 1 | | -2.50 | 25 | | | 1 | | 2 | | -2.11 | | | 29 | | | | | |  |  |
|  |  | 2 | | 3 | | -1.87 | 31 | | | 2 | | 7 | | -1.48 | | | 35 | | | | | |  |  |
|  |  | 3 | | 7 | | -1.45 | 36 | | | 3 | | 20 | | -0.85 | | | 42 | | | | | |  |  |
|  |  | 4 | | 14 | | -1.10 | 39 | | | 4 | | 40 | | -0.26 | | | 47 | | | | | |  |  |
|  |  | 5 | | 26 | | -0.65 | 44 | | | 5 | | 57 | | 0.14 | | | 52 | | | | | |  |  |
|  |  | 6 | | 41 | | -0.23 | 48 | | | 6 | | 78 | | 0.76 | | | 58 | | | | | |  |  |
|  |  | 7 | | 68 | | 0.46 | 55 | | | 7 | | 89 | | 1.21 | | | 62 | | | | | |  |  |
|  |  | 8 | | 88 | | 1.19 | 62 | | | 8 | | 99 | | 2.38 | | | 74 | | | | | |  |  |
|  | | Boys (n=136) | | | | | | Girls (n=149) | | | | | | | | | | | |  |  |  |  |  |
| Class 2 | | RS | | PR | | Z | T | | | RS | | PR | | Z | | | T | | | | | |  |  |
|  |  | 0 | | - | | - | - | | | 0 | | 0 | | - | | | - | | | | | |  |  |
|  |  | 1 | | - | | - | - | | | 1 | | 1 | | -2.47 | | | 25 | | | | | |  |  |
|  |  | 2 | | 0 | | - | - | | | 2 | | 3 | | -1.92 | | | 31 | | | | | |  |  |
|  |  | 3 | | 3 | | -1.90 | 31 | | | 3 | | 9 | | -1.35 | | | 36 | | | | | |  |  |
|  |  | 4 | | 7 | | -1.51 | 35 | | | 4 | | 16 | | -0.99 | | | 40 | | | | | |  |  |
|  |  | 5 | | 12 | | -1.16 | 38 | | | 5 | | 28 | | -0.67 | | | 44 | | | | | |  |  |
|  |  | 6 | | 27 | | -0.62 | 44 | | | 6 | | 46 | | -0.10 | | | 49 | | | | | |  |  |
|  |  | 7 | | 46 | | -0.11 | 49 | | | 7 | | 70 | | 0.53 | | | 55 | | | | | |  |  |
|  |  | 8 | | 75 | | 0.66 | 57 | | | 8 | | 94 | | 1.55 | | | 65 | | | | | |  |  |
| MOBAK 3-4 | | | | | | | | | | | | | | | | | | | | |  |  |  |  |
|  | | Boys (n=156) | | | | | | Girls (n=143) | | | | | | | | | | | |  |  |  |  |  |
|  | | RS | | PR | | Z | T | | | RS | | PR | | | Z | | | T | | | | | |  |
| Class 3 | | 0 | | 0 | | - | - | | | 0 | | 0 | | | - | | | - | | | | | |  |
|  |  | 1 | | 5 | | -1.69 | 25 | | | 1 | | 12 | | | -1.76 | | | 38 | | | | | |  |
|  |  | 2 | | 9 | | -0.34 | 31 | | | 2 | | 23 | | | -0.75 | | | 42 | | | | | |  |
|  |  | 3 | | 17 | | -0.96 | 36 | | | 3 | | 34 | | | -0.42 | | | 46 | | | | | |  |
|  |  | 4 | | 28 | | -0.57 | 39 | | | 4 | | 49 | | | -0.04 | | | 50 | | | | | |  |
|  |  | 5 | | 42 | | -0.20 | 44 | | | 5 | | 63 | | | 0.34 | | | 53 | | | | | |  |
|  |  | 6 | | 59 | | 0.22 | 48 | | | 6 | | 80 | | | 0.85 | | | 59 | | | | | |  |
|  |  | 7 | | 76 | | 0.71 | 55 | | | 7 | | 91 | | | 1.33 | | | 63 | | | | | |  |
|  |  | 8 | | 87 | | 1.13 | 62 | | | 8 | | 98 | | | 2.03 | | | 70 | | | | | |  |
|  | | Boys (n=149) | | | | | | Girls (n=167) | | | | | | | | | | | |  |  |  |  |  |
| Class 4 | | RS | | PR | | Z | T | | | RS | | PR | | | Z | | | T | | | | | |  |
|  |  | 0 | | 0 | | - | - | | | 0 | | 0 | | | - | | | - | | | | | |  |
|  |  | 1 | | 1 | | -2.21 | 28 | | | 1 | | 6 | | | -1.59 | | | 34 | | | | | |  |
|  |  | 2 | | 6 | | -1.61 | 34 | | | 2 | | 15 | | | -1.05 | | | 39 | | | | | |  |
|  |  | 3 | | 9 | | -1.35 | 36 | | | 3 | | 26 | | | -0.64 | | | 44 | | | | | |  |
|  |  | 4 | | 19 | | -0.88 | 41 | | | 4 | | 38 | | | -0.29 | | | 47 | | | | | |  |
|  |  | 5 | | 30 | | -0.53 | 45 | | | 5 | | 55 | | | 0.13 | | | 51 | | | | | |  |
|  |  | 6 | | 44 | | -0.15 | 48 | | | 6 | | 71 | | | 0.54 | | | 55 | | | | | |  |
|  |  | 7 | | 66 | | 0.50 | 54 | | | 7 | | 85 | | | 1.05 | | | 61 | | | | | |  |
|  |  | 8 | | 85 | | 1.04 | 60 | | | 8 | | 99 | | | 2.19 | | | 72 | | | | | |  |
| MOBAK 5-6 | | | | | | | | | | | | | | | | | | |  |  |  |  |  |  |
|  | Boys (n=108) | | | | | | | Girls (n=98) | | | | | | | | | | | |  |  |  |  |  |
|  | RS | | PR | | Z | | T | | RS | | PR | | Z | | | T | | | | | |  |  |  |
| Class 5 | 0 | | 0 | | - | | - | | 0 | | 0 | | - | | | - | | | | | |  |  |  |
|  | 1 | | 7 | | -1.51 | | 35 | | 1 | | 22 | | -0.78 | | | 42 | | | | | |  |  |  |
|  | 2 | | 14 | | -1.08 | | 39 | | 2 | | 39 | | -0.27 | | | 47 | | | | | |  |  |  |
|  | 3 | | 25 | | -0.67 | | 43 | | 3 | | 60 | | 0.25 | | | 52 | | | | | |  |  |  |
|  | 4 | | 38 | | -0.30 | | 47 | | 4 | | 76 | | 0.72 | | | 57 | | | | | |  |  |  |
|  | 5 | | 51 | | 0.01 | | 50 | | 5 | | 87 | | 1.08 | | | 61 | | | | | |  |  |  |
|  | 6 | | 64 | | 0.35 | | 53 | | 6 | | 94 | | 1.54 | | | 65 | | | | | |  |  |  |
|  | 7 | | 80 | | 0.86 | | 59 | | 7 | | 97 | | 1.87 | | | 69 | | | | | |  |  |  |
|  | 8 | | 95 | | 1.68 | | 67 | | 8 | | - | | - | | | - | | | | | |  |  |  |
|  | Boys (n=92) | | | | | | | Girls (n=100) | | | | | | | | | | | |  |  |  |  |  |
| Class 6 | RS | | PR | | Z | | T | | RS | | PR | | Z | | | T | | | | | |  |  |  |
|  | 0 | | 0 | | - | | - | | 0 | | 0 | | - | | | - | | | | | |  |  |  |
|  | 1 | | 6 | | -1.55 | | 34 | | 1 | | 34 | | -0.40 | | | 46 | | | | | |  |  |  |
|  | 2 | | 16 | | -1.01 | | 40 | | 2 | | 59 | | 0.22 | | | 52 | | | | | |  |  |  |
|  | 3 | | 27 | | -0.63 | | 44 | | 3 | | 69 | | 0.49 | | | 55 | | | | | |  |  |  |
|  | 4 | | 39 | | -0.29 | | 47 | | 4 | | 78 | | 0.76 | | | 58 | | | | | |  |  |  |
|  | 5 | | 59 | | 0.22 | | 52 | | 5 | | 89 | | 1.22 | | | 62 | | | | | |  |  |  |
|  | 6 | | 70 | | 0.52 | | 55 | | 6 | | 98 | | 2.05 | | | 70 | | | | | |  |  |  |
|  | 7 | | - | | - | | - | | 7 | | - | | - | | | - | | | | | |  |  |  |
|  | 8 | | 87 | | 1.11 | | 61 | | 8 | | 100 | | - | | | - | | | | | |  |  |  |

*Note:* PR=percentile rank; RS=raw scores; Z=z-score; T=T-value. Gray shaded: 40 ≤ T-value ≤ 60.

**
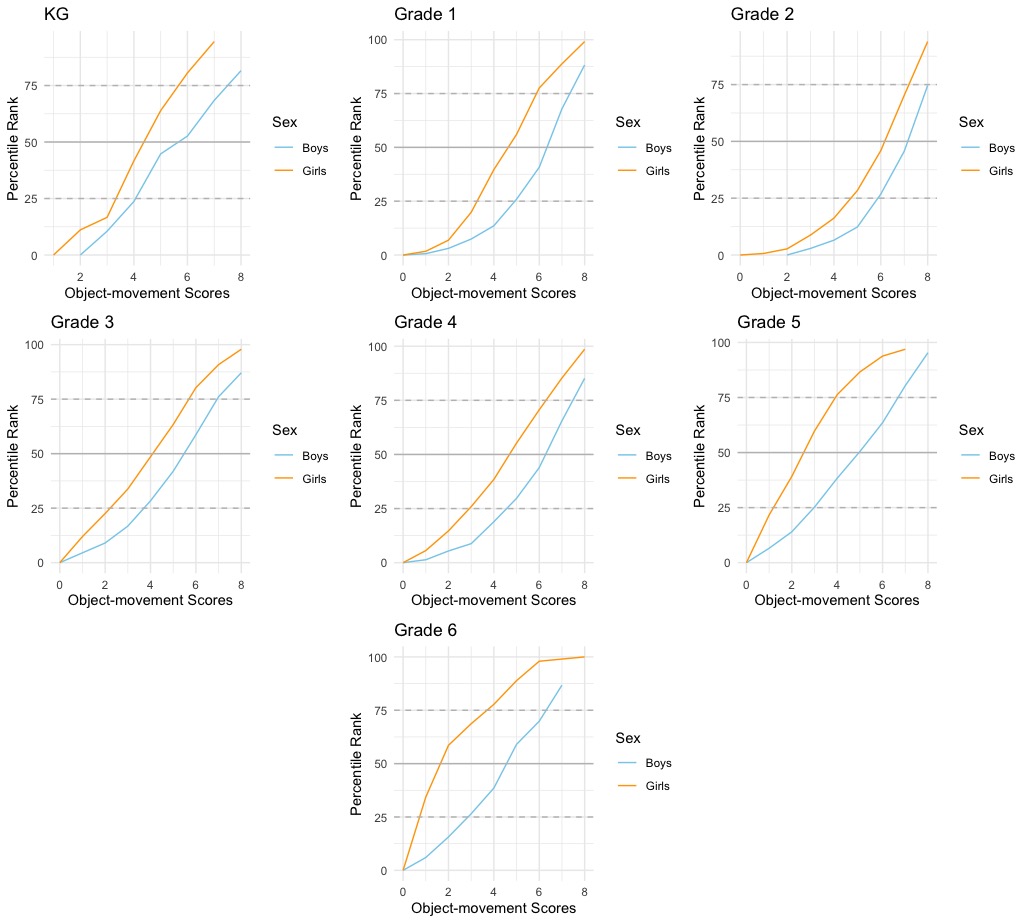
Figure S1.** Centile curves for the object-movement scores divided by grades and sex.

**Table S2.** Self-movement domain for KG, 1-2, 3-4 and 5-6 normative values (percentile ranks, z-score and t-values) divided by Grade and sex.

| MOBAK KG | | | | | | | | | | | | | | | | | | | | |  |  |  |  |  |  |
| --- | --- | --- | --- | --- | --- | --- | --- | --- | --- | --- | --- | --- | --- | --- | --- | --- | --- | --- | --- | --- | --- | --- | --- | --- | --- | --- |
|  | Boys (n=39) | | | | | | | | | Girls (n=37) | | | | | | | | | | | | |  |  |  |  |
|  | RS | PR | | | Z | | | | T | | | | | RS | | PR | | Z | | T | | | | | |  |
| Class KG | 0 | 0 | | | - | | | | - | | | | | 0 | | - | | - | | - | | | | | |  |
|  | 1 | 3 | | | 1.94 | | | | 31 | | | | | 1 | | 0 | | - | | - | | | | | |  |
|  | 2 | 11 | | | -1.25 | | | | 37 | | | | | 2 | | 3 | | -1.91 | | 31 | | | | | |  |
|  | 3 | 13 | | | -1.12 | | | | 39 | | | | | 3 | | 6 | | -1.59 | | 34 | | | | | |  |
|  | 4 | 24 | | | -0.72 | | | | 43 | | | | | 4 | | 22 | | -0.76 | | 42 | | | | | |  |
|  | 5 | 58 | | | 0.20 | | | | 52 | | | | | 5 | | 33 | | -0.43 | | 46 | | | | | |  |
|  | 6 | 66 | | | 0.41 | | | | 54 | | | | | 6 | | 67 | | 0.43 | | 54 | | | | | |  |
|  | 7 | 84 | | | 1.00 | | | | 60 | | | | | 7 | | 83 | | 0.97 | | 60 | | | | | |  |
|  | 8 | 100 | | | - | | | | - | | | | | 8 | | 94 | | 1.59 | | 66 | | | | | |  |
| MOBAK 1-2 | | | | | | | | | | | | | | | | | | | | | | |  | | | |
|  | Boys (n=163) | | | | | | | | | Girls (n=117) | | | | | | | | | | | | |  |  |  |  |
|  | RS | PR | | Z | | | T | | | | | RS | | | PR | | Z | | T | | | | | |  |  |
| Class 1 | 0 | 0 | | - | | | - | | | | | 0 | | | 0 | | - | | - | | | | | |  |  |
|  | 1 | 1 | | -2.50 | | | 25 | | | | | 1 | | | 1 | | -2.38 | | 26 | | | | | |  |  |
|  | 2 | 3 | | -1.87 | | | 31 | | | | | 2 | | | 3 | | -1.81 | | 32 | | | | | |  |  |
|  | 3 | 10 | | -1.29 | | | 37 | | | | | 3 | | | 9 | | -1.31 | | 37 | | | | | |  |  |
|  | 4 | 21 | | -0.81 | | | 42 | | | | | 4 | | | 18 | | -0.91 | | 41 | | | | | |  |  |
|  | 5 | 33 | | -0.45 | | | 46 | | | | | 5 | | | 37 | | -0.33 | | 47 | | | | | |  |  |
|  | 6 | 54 | | 0.17 | | | 51 | | | | | 6 | | | 57 | | 0.17 | | 52 | | | | | |  |  |
|  | 7 | 81 | | 0.85 | | | 59 | | | | | 7 | | | 80 | | 0.85 | | 58 | | | | | |  |  |
|  | 8 | 93 | | 1.82 | | | 64 | | | | | 8 | | | 97 | | 1.82 | | 68 | | | | | |  |  |
|  | Boys (n=136) | | | | | | | | | Girls (n=149) | | | | | | | | | | | | |  |  |  |  |
| Class 2 | RS | PR | | | Z | | | T | | | | RS | | | PR | | Z | | T | | | | |  |  |  |
|  | 0 | 0 | | | - | | | - | | | | 0 | | | - | | - | | - | | | | |  |  |  |
|  | 1 | 1 | | | -2.44 | | | 26 | | | | 1 | | | 0 | | - | | - | | | | |  |  |  |
|  | 2 | 2 | | | -2.18 | | | 28 | | | | 2 | | | 3 | | -1.92 | | 31 | | | | |  |  |  |
|  | 3 | 7 | | | -1.46 | | | 35 | | | | 3 | | | 5 | | -1.67 | | 33 | | | | |  |  |  |
|  | 4 | 17 | | | -0.97 | | | 40 | | | | 4 | | | 11 | | -1.20 | | 38 | | | | |  |  |  |
|  | 5 | 33 | | | -0.43 | | | 46 | | | | 5 | | | 16 | | -1.01 | | 40 | | | | |  |  |  |
|  | 6 | 54 | | | 0.09 | | | 51 | | | | 6 | | | 31 | | -0.49 | | 45 | | | | |  |  |  |
|  | 7 | 76 | | | 0.71 | | | 57 | | | | 7 | | | 55 | | 0.14 | | 51 | | | | |  |  |  |
|  | 8 | 93 | | | 1.51 | | | 65 | | | | 8 | | | 79 | | 0.83 | | 58 | | | | |  |  |  |
| MOBAK 3-4 | | | | | | | | | | | | | | | | | | | | | | |  |  |  |  |
|  | Boys (n=156) | | | | | | | | | | Girls (n=143) | | | | | | | | | | | |  |  |  |  |
|  | RS | | PR | | | Z | | | T | | | | RS | | | PR | | Z | | T | | | | |  |  |
| Class 3 | 0 | | 0 | | | - | | | - | | | | 0 | | | 0 | | - | | - | | | | |  |  |
|  | 1 | | 8 | | | -1.42 | | | 35 | | | | 1 | | | 6 | | -1.53 | | 35 | | | | |  |  |
|  | 2 | | 17 | | | -0.94 | | | 41 | | | | 2 | | | 14 | | -1.08 | | 39 | | | | |  |  |
|  | 3 | | 39 | | | -0.29 | | | 47 | | | | 3 | | | 33 | | -0.44 | | 46 | | | | |  |  |
|  | 4 | | 55 | | | 0.14 | | | 51 | | | | 4 | | | 46 | | -0.11 | | 49 | | | | |  |  |
|  | 5 | | 72 | | | 0.57 | | | 56 | | | | 5 | | | 64 | | 0.36 | | 54 | | | | |  |  |
|  | 6 | | 85 | | | 1.01 | | | 60 | | | | 6 | | | 73 | | 0.62 | | 56 | | | | |  |  |
|  | 7 | | 92 | | | 1.42 | | | 64 | | | | 7 | | | 88 | | 1.18 | | 61 | | | | |  |  |
|  | 8 | | 97 | | | 1.85 | | | 68 | | | | 8 | | | 95 | | 1.65 | | 67 | | | | |  |  |
|  | Boys (n=149) | | | | | | | | | | Girls (n=167) | | | | | | | | | | | |  |  |  |  |
| Class 4 | RS | | PR | | | Z | | | T | | | | RS | | | PR | | Z | | T | | | | |  |  |
|  | 0 | | 0 | | | - | | | - | | | | 0 | | | 0 | | - | | - | | | | |  |  |
|  | 1 | | 5 | | | -1.61 | | | 34 | | | | 1 | | | 3 | | -1.91 | | 31 | | | | |  |  |
|  | 2 | | 11 | | | -1.20 | | | 38 | | | | 2 | | | 10 | | -1.29 | | 37 | | | | |  |  |
|  | 3 | | 29 | | | -0.55 | | | 44 | | | | 3 | | | 22 | | -0.78 | | 42 | | | | |  |  |
|  | 4 | | 41 | | | -0.22 | | | 48 | | | | 4 | | | 35 | | -0.39 | | 46 | | | | |  |  |
|  | 5 | | 62 | | | 0.31 | | | 53 | | | | 5 | | | 52 | | 0.06 | | 51 | | | | |  |  |
|  | 6 | | 72 | | | 0.59 | | | 56 | | | | 6 | | | 71 | | 0.54 | | 55 | | | | |  |  |
|  | 7 | | 86 | | | 1.07 | | | 61 | | | | 7 | | | 85 | | 1.05 | | 61 | | | | |  |  |
|  | 8 | | 93 | | | 1.49 | | | 65 | | | | 8 | | | 95 | | 1.66 | | 67 | | | | |  |  |
| MOBAK 5-6 | | | | | | | | | | | | | | | | | | | | | |  |  |  |  |  |
|  | Boys (n=108) | | | | | | | | | | Girls (n=98) | | | | | | | | | | | |  |  |  |  |
|  | RS | | PR | | | Z | | | T | | | | RS | | | PR | | Z | | T | | | | |  |  |
| Class 5 | 0 | | 0 | | | - | | | - | | | | 0 | | | 0 | | - | | - | | | | |  |  |
|  | 1 | | 10 | | | -1.27 | | | 37 | | | | 1 | | | 11 | | -1.21 | | 38 | | | | |  |  |
|  | 2 | | 23 | | | -0.73 | | | 43 | | | | 2 | | | 18 | | -0.93 | | 41 | | | | |  |  |
|  | 3 | | 34 | | | -0.42 | | | 46 | | | | 3 | | | 31 | | -0.50 | | 45 | | | | |  |  |
|  | 4 | | 50 | | | -0.01 | | | 50 | | | | 4 | | | 48 | | -0.04 | | 50 | | | | |  |  |
|  | 5 | | 67 | | | 0.45 | | | 54 | | | | 5 | | | 65 | | 0.38 | | 54 | | | | |  |  |
|  | 6 | | 78 | | | 0.76 | | | 58 | | | | 6 | | | 73 | | 0.62 | | 56 | | | | |  |  |
|  | 7 | | 87 | | | 1.12 | | | 61 | | | | 7 | | | 87 | | 1.11 | | 61 | | | | |  |  |
|  | 8 | | 93 | | | 1.44 | | | 64 | | | | 8 | | | 95 | | 1.63 | | 66 | | | | |  |  |
|  | Boys (n=92) | | | | | | | | | | Girls (n=100) | | | | | | | | | | | |  |  |  |  |
| Class 6 | RS | | PR | | | Z | | | T | | | | RS | | | PR | | Z | | T | | | | |  |  |
|  | 0 | | 0 | | | - | | | - | | | | 0 | | | 0 | | - | | - | | | | |  |  |
|  | 1 | | 4 | | | -1.80 | | | 32 | | | | 1 | | | 6 | | -1.55 | | 35 | | | | |  |  |
|  | 2 | | 18 | | | -0.91 | | | 41 | | | | 2 | | | 14 | | -1.07 | | 39 | | | | |  |  |
|  | 3 | | 45 | | | -0.14 | | | 49 | | | | 3 | | | 23 | | -0.73 | | 43 | | | | |  |  |
|  | 4 | | 57 | | | 0.17 | | | 52 | | | | 4 | | | 36 | | -0.35 | | 47 | | | | |  |  |
|  | 5 | | 71 | | | 0.56 | | | 56 | | | | 5 | | | 48 | | -0.04 | | 50 | | | | |  |  |
|  | 6 | | 77 | | | 0.74 | | | 57 | | | | 6 | | | 60 | | 0.24 | | 52 | | | | |  |  |
|  | 7 | | 92 | | | 1.38 | | | 64 | | | | 7 | | | 86 | | 1.07 | | 61 | | | | |  |  |
|  | 8 | | 96 | | | 1.80 | | | 68 | | | | 8 | | | 93 | | 1.47 | | 65 | | | | |  |  |

*Note:* PR=percentile rank; RS=raw scores; Z=z-score; T=T-value. Gray shaded: 40 ≤ T-value ≤ 60.

**
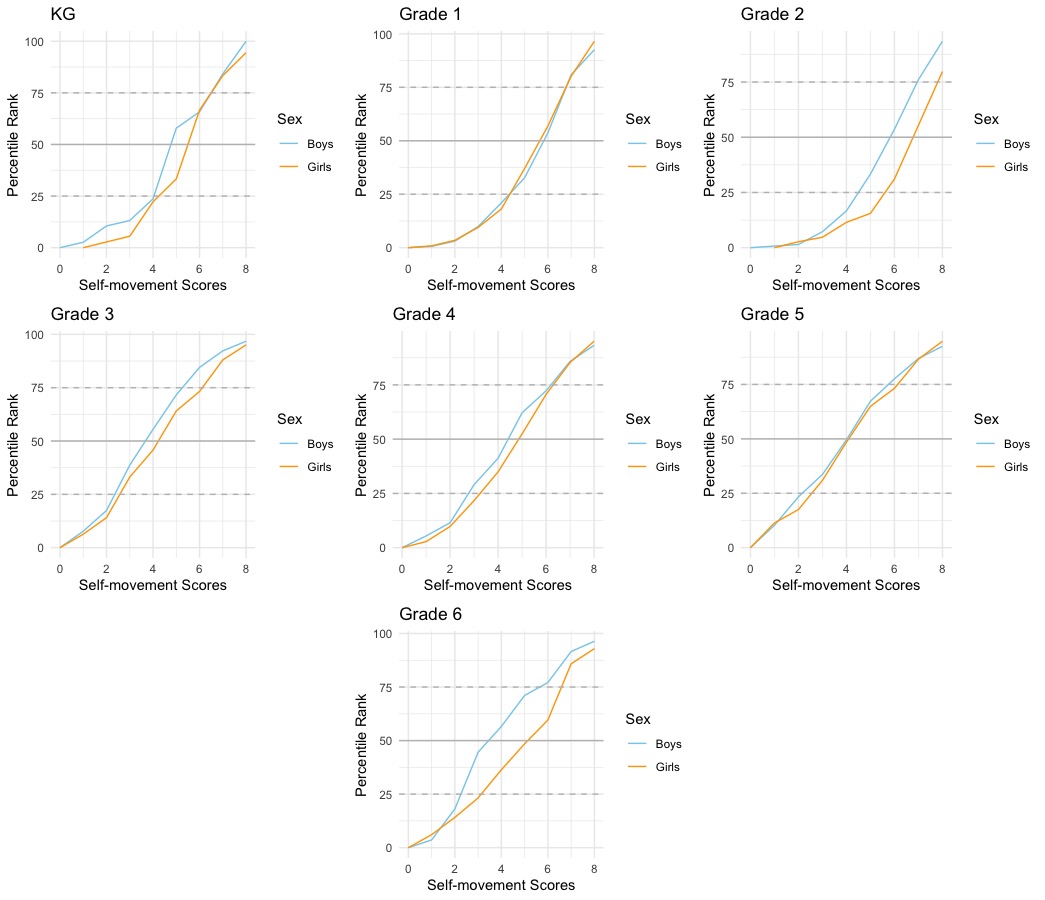
Figure S2.** Centile curves for the object-movement scores divided by grades and sex.

**
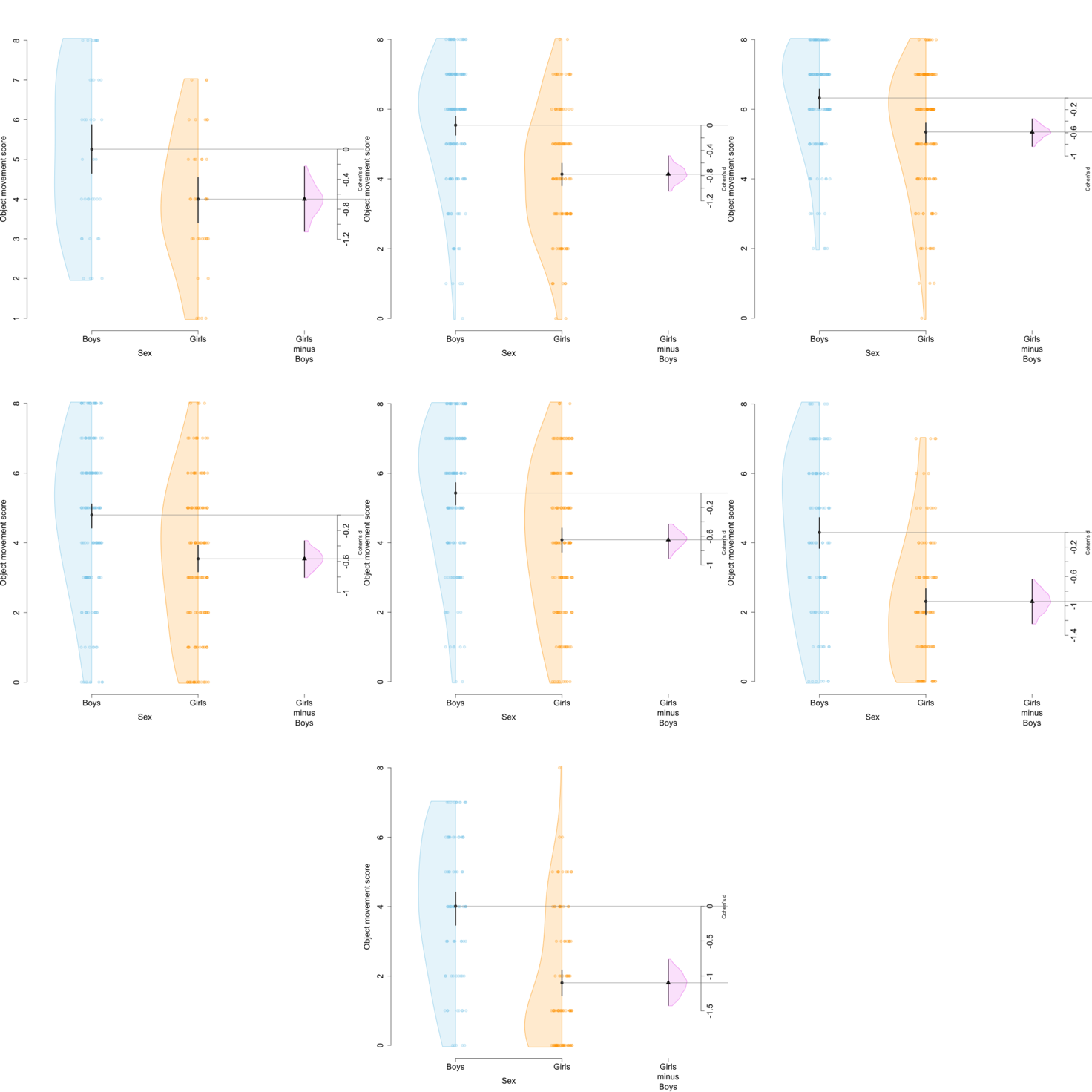
**

**Figure S3.** Object-movement score differences are displayed by Gardner-Altman plots which show a representation of observed values by the two-group comparison (MHOO vs. MUOO), a bootstrap effect size (Cohen’s d) estimation, mean and 95% confidence interval. Two-sided P values from analysis of covariance evaluating the differences between groups adjusting for sex and age.

**
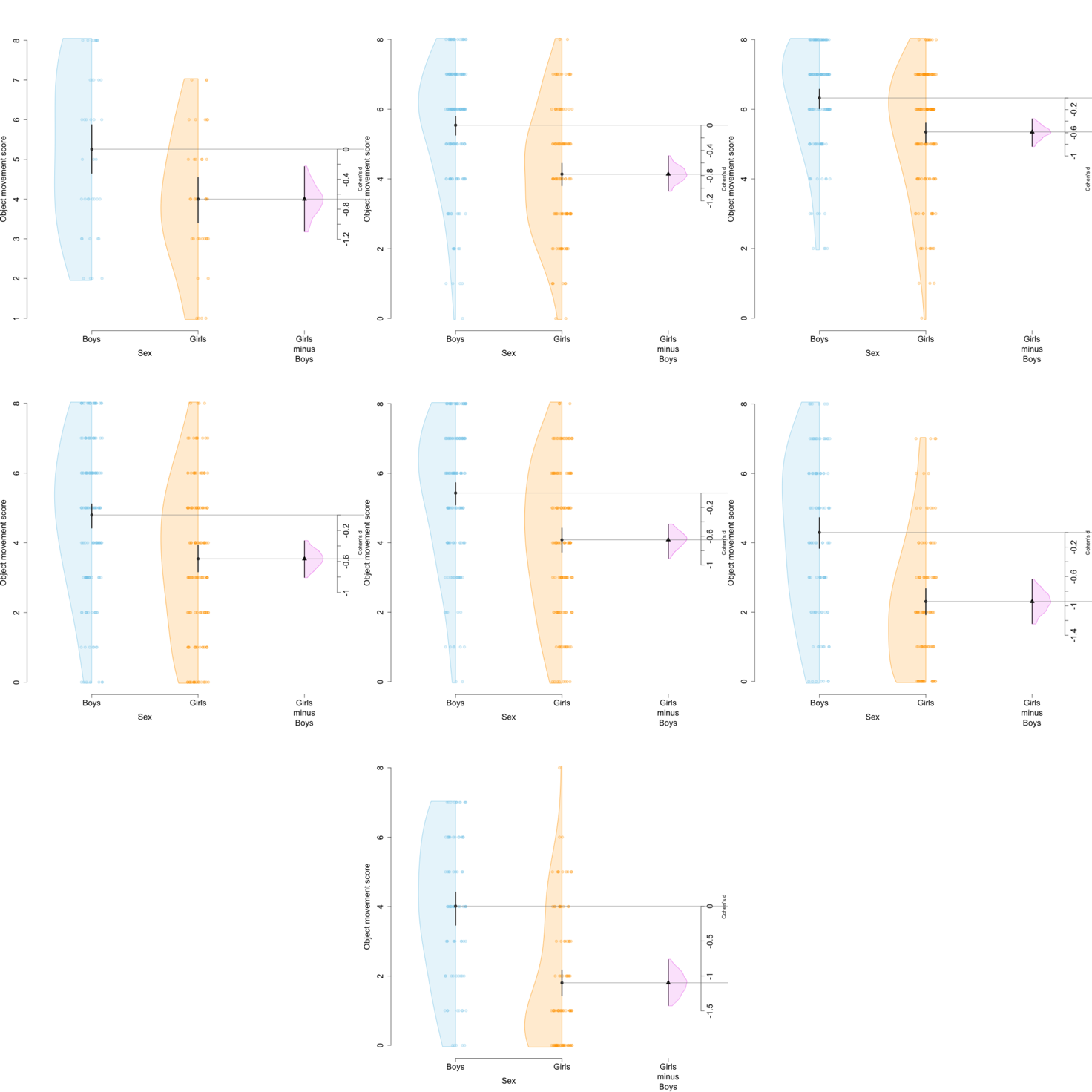
**

**Figure S4.** Self-movement score differences are displayed by Gardner-Altman plots which show a representation of observed values by the two-group comparison (MHOO vs. MUOO), a bootstrap effect size (Cohen’s d) estimation, mean and 95% confidence interval. Two-sided P values from analysis of covariance evaluating the differences between groups adjusting for sex and age.


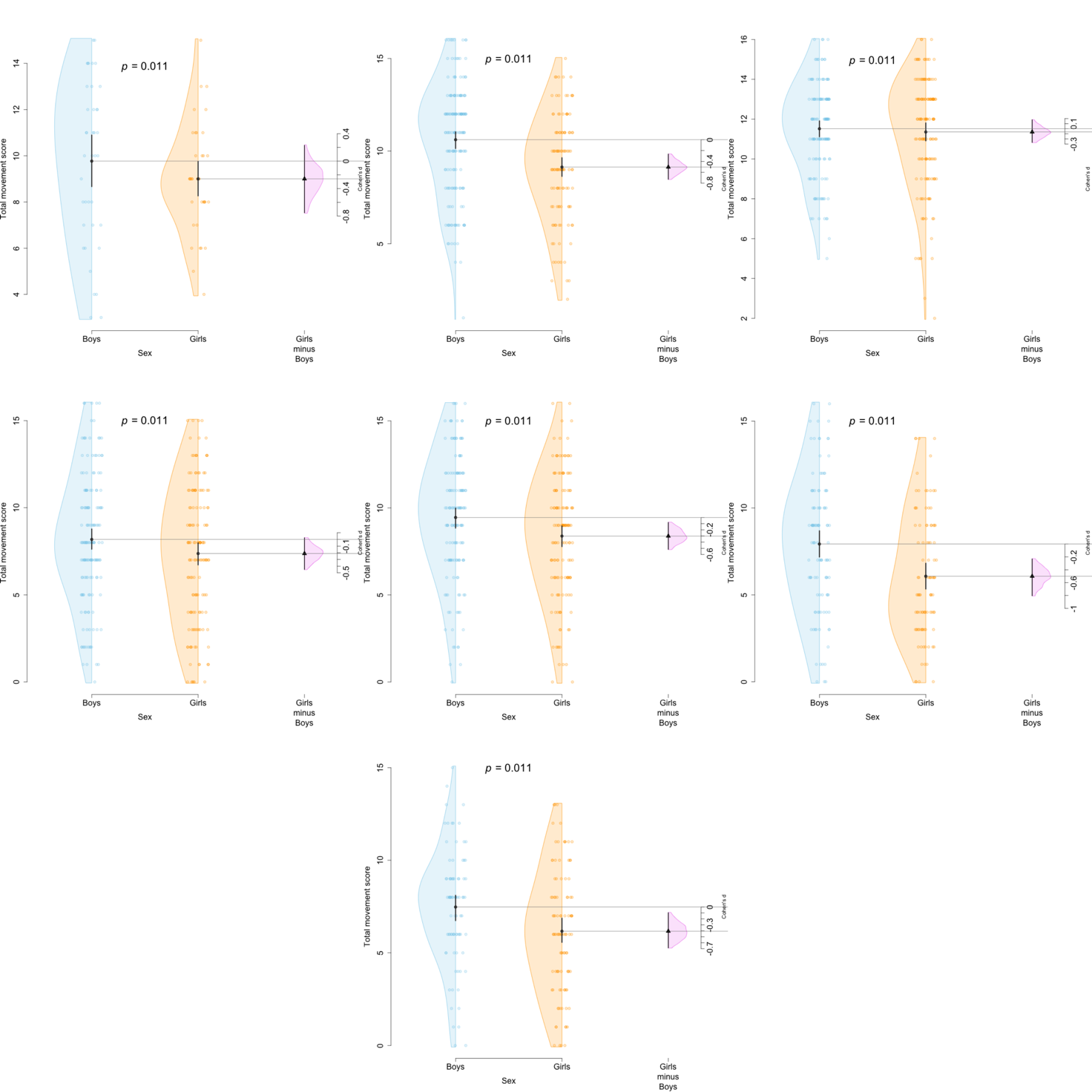


**Figure S5.** Total MOBAK score differences are displayed by Gardner-Altman plots which show a representation of observed values by the two-group comparison (MHOO vs. MUOO), a bootstrap effect size (Cohen’s d) estimation, mean and 95% confidence interval. Two-sided P values from analysis of covariance evaluating the differences between groups adjusting for sex and age.

*Summary of MOBAK battery items*

| **Competence** | **Item** | **Section** | **Description** |
| --- | --- | --- | --- |
| **Object movement** | throwing | MOBAK KG | *Throw a juggling balls six rounders from a scratch line marked 1.5m in front of the target placed at 1.1 m height on a wall* |
|  |  | MOBAK 1-2 | *Throw juggling balls six rounders from a scratch line marked 2.0m in front of the target placed at 1.3 m height on a wall* |
|  |  | MOBAK 3-4 | *Throw juggling balls six rounders from a scratch line marked 3.0m in front of the target placed at 1.3 m height on a wall* |
|  |  | MOBAK 5-6 | *Throw juggling balls six rounders from a scratch line marked 3.5m in front of the target placed at 1.3 m height on a wall* |
|  | catching | MOBAK KG | *Catch the ball after the bounce about 1.1m up on the floor before it falls to the ground* |
|  |  | MOBAK 1-2 | *Catch a rubber ball after a bounce on the ground.* |
|  |  | MOBAK 3-4 | *Throw a ball in the air behind the first line, follow the ball and catch it before it falls to the ground behind the second line.* |
|  |  | MOBAK 5-6 | *Throws a tennis ball at the wall and catch it directly when still in the air.* |
|  | bouncing | MOBAK KG | *Bounce continuously the ball to the floor with both hands and catch it again without losing the ball* |
|  |  | MOBAK 1-2 | *Bounce a a basketball ball (N°3) through the corridor (5.0m x 1.0m)* |
|  |  | MOBAK 3-4 | *Bounce a basketball ball (N°3) through the corridor (7.0m x 1.4m) with four obastacles.* |
|  |  | MOBAK 5-6 | *Bounce a basketball ball (N°6) quickly back and forth through an obstacle course with obstacle (8.0m x 1.1m)* |
|  | dribbling | MOBAK KG | *Dribble a football ball along a corridor until the finish line without losing the ball* |
|  |  | MOBAK 1-2 | *Dribble a football ball (N°4) along a corridor (5.0m x 1.0m) until the finish line without losing the ball* |
|  |  | MOBAK 3-4 | *Dribble a football ball (N°4) along a corridor (7.5m x 1.4m) with obstacles until the turning point and then back again without losing the ball* |
|  |  | MOBAK 5-6 | *Dribble a football ball (N°4) quickly back and forth through an obstacle course (8.0m x 1.1m).* |
| **Self-movement** | balancing | MOBAK KG | *Balances forward over a reversed long bench from the first marking to the second marking, touch it and then balances backwards back to the first marking.* |
|  |  | MOBAK 1-2 | *Balance forwards and backwards across a see-sawing long bench* |
|  |  | MOBAK 3-4 | *Balance forwards and backwards over the long bench with 2 obstacles of 6cm high* |
|  |  | MOBAK 5-6 | *Balance forwards and backwards over the long bench with 2 obstacles of 12cm high* |
|  | rolling | MOBAK KG | *From a squat down position, perform a roll forward on the downwardly inclined plane and come to a standstill on feet in one flow* |
|  |  | MOBAK 1-2 | From a squat position, perform *a roll forward*, with your hands placed on the mat. |
|  |  | MOBAK 3-4 | *Performs a roll forward over 2 longitudinal box pair* |
|  |  | MOBAK 5-6 | *From a two-legged stand position, perform a perform a diving forward roll over the Box and come to the starting position* |
|  | *Jumping* | MOBAK KG | *From two-legged stand position behind the starting line continuously jump on one leg until the end line. Afterwards turn and jump on the other leg back to the starting line.* |
|  |  | MOBAK 1-2 | *Jump continuously across 4 tiles placed onthe floor, between the tiles standing on one foot while beneath the tiles jumping with straddled legs.* |
|  |  | MOBAK 3-4 | *Skip the rope without interruption for 20 seconds in place* |
|  |  | MOBAK 5-6 | *Skip the rope 20s with changing rhythms at 10s* |
|  | Running | *MOBAK KG* | *Run forward in a corridor to the wall touching it and run backwards to the start line for two times* |
|  |  | *MOBAK 1-2* | *Run fluently sideways from the first marker to the second on a 3.0m line* |
|  |  | *MOBAK 3-4* | *Run forward and sideways in a rectangular space (2.0m x 4.0m)* |
|  |  | *MOBAK 5-6* | *Run forward and sideaway in a square space (4.0m x 4.0m) with three steps in each hoop placed on the floor when running forward* |
